# Traumatic Bilateral Perched Facet of the Thoracic Spine With Associated Vertebral Fracture: A Case Report

**DOI:** 10.7759/cureus.88187

**Published:** 2025-07-17

**Authors:** Seiya Watanabe, Kazuo Nakanishi, Yoshihisa Sugimoto, Tomoyuki Noda, Shigeru Mitani

**Affiliations:** 1 Orthopaedics, Kawasaki Medical School, Okayama, JPN; 2 Orthopaedic Surgery, Kawasaki Medical School General Medical Center, Okayama, JPN

**Keywords:** dislocation, perched facet, posterior fusion, thoracic vertebra, tlics

## Abstract

Bilateral perched facets of the cervical spine are relatively common in trauma cases; however, similar injuries in the thoracic spine are exceedingly rare due to the inherent stability provided by the rib cage and associated ligamentous structures. We report a rare case of bilateral perched facets at the T10/11 level in a 17-year-old female gymnast who fell from a height of 3 m during training. She presented with severe back pain but no neurological deficits and was ambulatory on arrival. Imaging revealed bilateral perched facets without fracture of facet joints or spinal cord compression, but with evidence of posterior ligamentous complex injury. The Thoracolumbar Injury Classification and Severity Score was 7, indicating instability. Hyperextension reduction was successfully performed, followed by posterior spinal fusion with instrumentation due to persistent instability. The patient recovered well postoperatively and was discharged with no neurological deficits. This case highlights the importance of considering unstable thoracic spine injuries, even in the absence of neurological symptoms, following high-energy trauma. Accurate diagnosis and timely surgical intervention are essential to prevent potentially severe outcomes.

## Introduction

Bilateral perched facets of the cervical spine without associated fractures are relatively common traumatic injuries. A perched facet is defined as a condition in which the inferior articular process of the superior vertebra is perched directly on top of the superior articular process of the inferior vertebra [1]. In contrast, bilateral perched facets of the thoracic spine, which are stabilized by the rib cage, are extremely rare and there were six cases of this condition that were found in the literature [2-4]. Four of the six cases showed neurological abnormalities in the lower limbs. Thoracic perched facets are thought to be caused by excessive flexion of the thoracic spine due to high-energy trauma, such as falls from heights or traffic accidents [2-4]. Dislocations are most likely to occur in the 10th to 12th thoracic vertebrae [5]. However, the regions where perched facets are likely to occur are not clear. We report a case of traumatic bilateral perched facet without fracture of facet joints and without neurological abnormalities in the lower limbs.

## Case presentation

**Figure 1:**
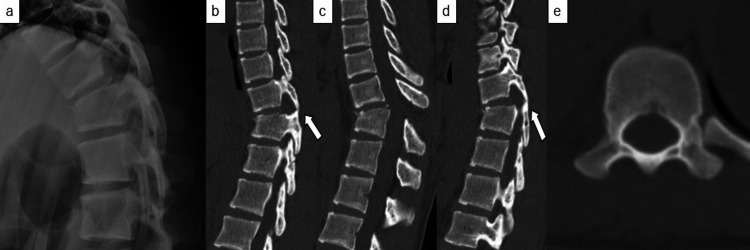
Preoperative imaging examination (a) Preoperative plain thoracic X-ray. There was a compression wedge fracture of T11. (b) Preoperative plain thoracic computed tomography (CT) of the left facet joint. (c) Preoperative plain thoracic CT in the sagittal plane. There was a compression wedge fracture of T11. (d) Preoperative plain thoracic CT of the right facet joint. (e) Preoperative plain thoracic CT in the axial plane

Herein, we report a case of traumatic bilateral thoracic perched facets in a 17-year-old female gymnast with no significant medical history. She presented with severe back pain after falling from a height of 3 m while practicing on a horizontal bar. There were no head and appendicular injuries. Physical examination revealed back pain but no lower extremity muscle weakness, and she was able to ambulate independently. Thoracic spine radiography and sagittal computed tomography (CT) revealed bilateral perched facets at the T10/11 level with T11 vertebrae fracture (Figure 1).

**Figure 2:**
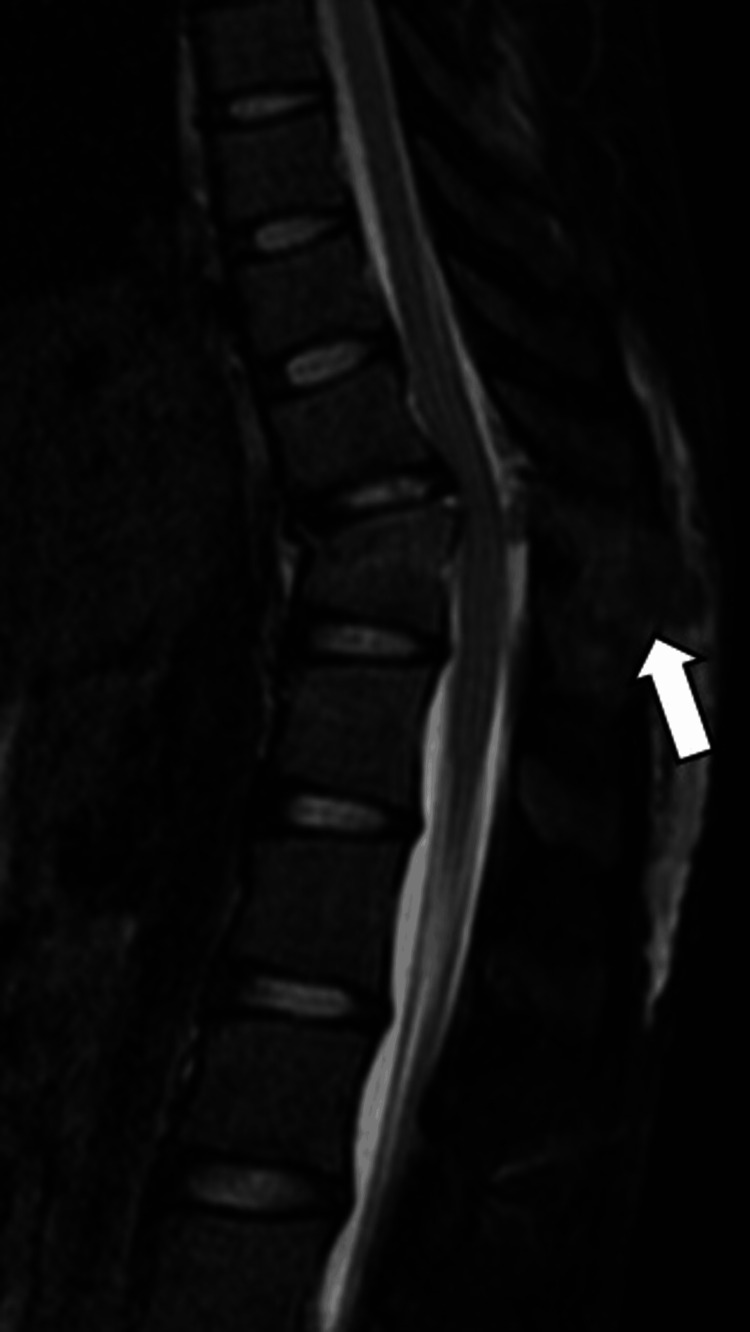
Preoperative plain thoracic magnetic resonance imaging Preoperative plain thoracic magnetic resonance imaging showing no signal intensity changes in the spinal cord, with evidence of posterior ligamentous complex injury (white arrow). There was a compression wedge fracture of T11.

In the AO (Arbeitsgemeinschaft für Osteosynthesefragen) Spine classification, the injury type was C, neurological abnormalities were N0, and clinical modifiers were M1. Magnetic resonance imaging was not indicative of spinal cord injury; however, signal changes in the posterior ligamentous structures suggested a posterior component injury. There was also a compression wedge fracture of T11. The Thoracolumbar Injury Classification and Severity Score was 7, indicating significant instability: 4 points for injury morphology (dislocation), 3 for posterior ligamentous complex injury, and 0 for neurological deficits (Figure 2).

**Figure 3:**
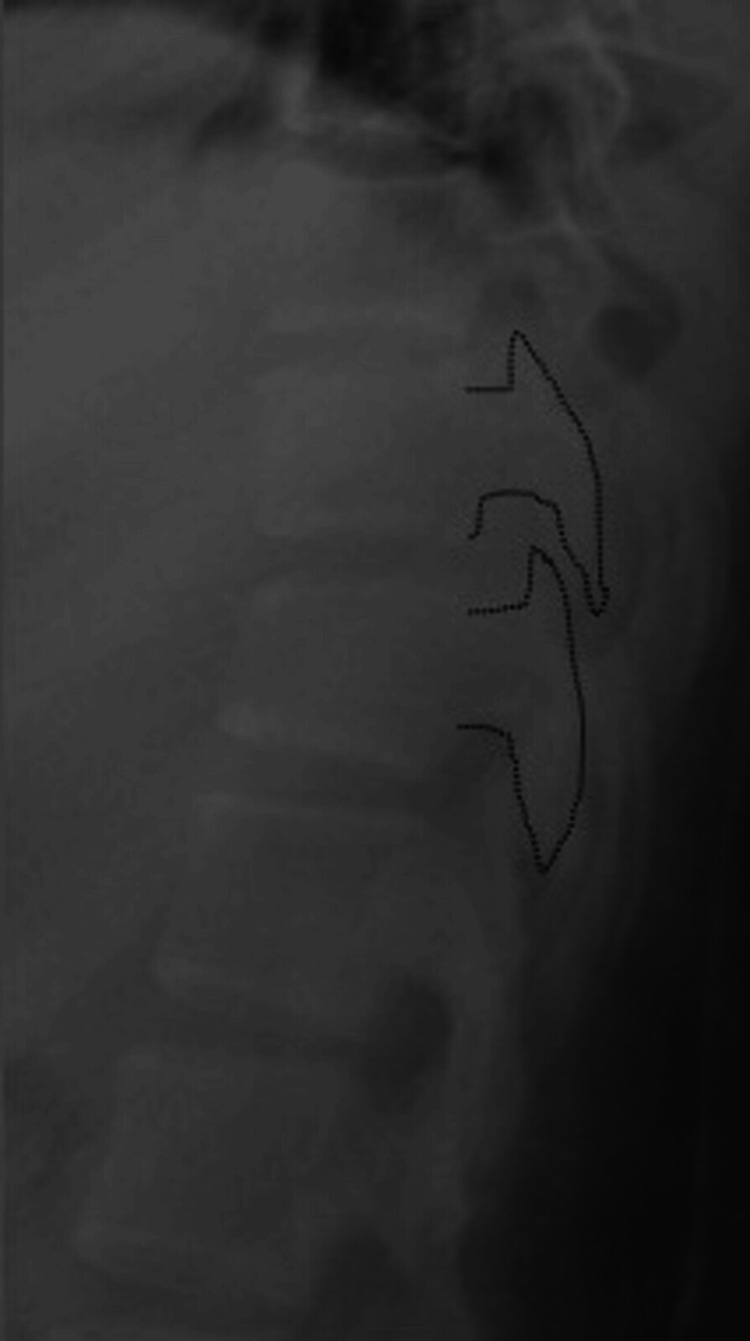
X-ray after closed reduction Reduction of facet joint dislocation achieved by hyperextension.

Based on the imaging findings, it is inferred that the patient sustained a fall from a significant height, resulting in hyperflexion of the thoracic spine and a compression fracture at the T11 vertebral level. In addition, a concomitant rotational force appears to have led to a perched facet. We have determined that this is AO-Magerl classification C1 and consider it to be highly unstable. The patient was admitted and underwent immediate hyperextension reduction. After maintaining the hyperextended position for 3 h, successful reduction of the dislocation was achieved (Figure 3).

**Figure 4:**
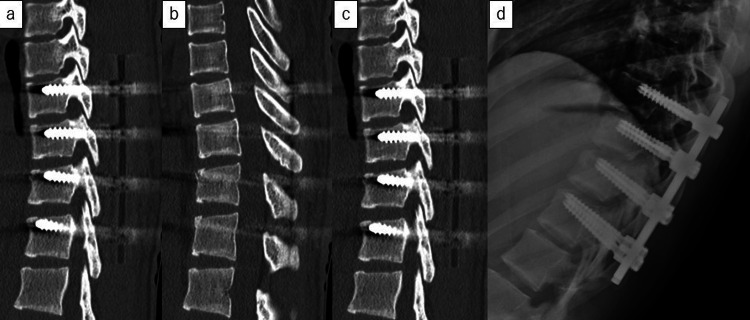
Postoperative imaging examination (a) Postoperative plain thoracic computed tomography (CT) of the left facet joint. (b) Postoperative plain thoracic CT in the sagittal plane. (c) Postoperative plain thoracic CT of the right facet joint. (d) Postoperative plain thoracic X-ray.

However, surgical intervention was deemed necessary due to instability. Posterior spinal fusion with instrumentation (two levels above and below the affected segment) was performed in the prone position (Figure 4).

The operative time was 70 min, with an estimated blood loss of 10 g. Postoperatively, the patient was fitted with a soft brace and was able to ambulate independently before discharge. From now on, we will continue to examine the patient as an outpatient until bone union is confirmed. Once the bone union is confirmed, we plan to perform the removal of the implants.

## Discussion

The thoracic spine is considered more stable than the cervical and lumbar spine [6]. The higher risk of cervical spine dislocation is due to the relatively small facet joints and orientation of approximately 45° to the horizontal plane [7]. In contrast, the facet joints of the thoracic spine are larger and more vertically oriented, leading to greater stability. Additional stabilizing factors include the restraining effect of the rib cage and costotransverse ligaments, sagittal orientation of the facet joints, and presence of robust posterior longitudinal and ligamentum flavum complexes [8]. Manaster and Osborn classified thoracolumbar facet dislocations into three major types: (1) anterior dislocation of the vertebral body with anterior displacement of the inferior articular process; (2) lateral dislocation of the vertebral body with lateral displacement of the inferior articular process; and (3) facet subluxation without vertebral dislocation, characterized by significant superior displacement of the articular facet [9]. The present case falls into the third category but does not represent a complete dislocation, classifying it strictly as a perched facet, which typically occurs in high-energy trauma scenarios such as motor vehicle accidents. Owing to the relatively narrow spinal canal in the thoracic region, complete spinal cord injury with resultant paraplegia occurs in >80% of cases following dislocation [8]. Consequently, bilateral perched facets of the thoracic spine without neurological deficits are considered exceedingly rare. In cases such as ours, where the patient presents without neurological deficits and is ambulatory, there is a risk of misdiagnosing the injury as a minor contusion, leading to inadequate treatment. Proper diagnosis and timely management are crucial to prevent potentially catastrophic neurological sequelae. The surgical treatment method for thoracic dislocation fractures including perched facets has been reported to be stabilized using pedicle screws through a posterior approach [5,10-12]. Previous reports of thoracic perched facets have all undergone surgical treatment, with no cases treated conservatively [2-4]. As with previous reports, this case also demonstrated favorable outcomes following posterior fixation surgery.

## Conclusions

We reported an extremely rare case of bilateral perched facets of the thoracic spine without fracture of facet joints or neurological deficits. Thoracic dislocation is thought to occur due to excessive flexion. In this case, we think that excessive flexion of the thoracic spine occurred due to the accompanying compression fracture of T11. This case highlights the importance of recognizing such injuries, even in the absence of severe neurological symptoms, especially given the inherent stability of the thoracic spine and the risk of underdiagnosis. Timely radiographic evaluation and appropriate classification are essential to guide management. Despite the absence of neurological impairment, the significant instability necessitated surgical stabilization. Clinicians should maintain a high index of suspicion for spinal instability in patients with high-energy thoracic trauma, as early diagnosis and intervention are critical to prevent potentially devastating outcomes.
